# Bipolar plasma vaporization – an innovative intramural ureter detachment method during nephroureterectomy

**Published:** 2012-06-18

**Authors:** P Geavlete, R Multescu, B Geavlete, D Georgescu, C Moldoveanu

**Affiliations:** “Sf. Ioan” Clinical Emergency Hospital, Department of Urology, Bucharest, Romania

**Keywords:** urothelial upper urinary-tract carcinoma, pluck technique

## Abstract

**Introduction:**Nephroureterectomy with perimeatal cystectomy is still the gold standard in the treatment of urothelial upper urinary-tract carcinoma (UUTC). Ureteral endoscopic surgery was proposed as a complementary first step in nephroureterectomy, in order to obviate the low abdominal incision. Our goal was to establish the value of a novel method of endoscopic distal ureteral management in on step nephroureterectomy for UUTC: pluck technique by using bipolar plasma vaporization.

**Materials and Methods:**During the last two years, we performed nephroureterectomy with pluck transurethral detachment of the intramural ureter by using bipolar plasma vaporization in 42 cases with UUTC (pTa in 16 cases, pT1 in 10 cases, pT2 in 9 cases, pT3 in 7 cases). The tumor was pyelocaliceal in 34 cases, ureteral in 7 cases, and both ureteral and pyelocaliceal in 1 case. The follow-up was performed by cystoscopy with urinary cytology, ultrasonography, intravenous urography and CT. The mean follow-up was of 14 months (range 8 to 26 months).

**Results:**All procedures were completed successfully. The complications rate was of 4.8%: 2 cases of hematuria, one imposing an endoscopic approach and another one treated conservatively. During follow-up, 6 patients had bladder recurrences, 1 had renal fossa tumors and 1 had secondary lymph-node invasion. The disease-specific mortality rate was of 4.8%.

**Conclusions:**The endoscopic approach of the terminal ureter with bipolar plasma vaporization as part of one-step nephroureterectomy is a safe, facile and effective method offering good oncological results.

## Introduction

Nephroureterectomy with perimeatal cystectomy is still regarded by many as the gold-standard therapy for upper urinary tract urothelial tumors. However, this is an extensive surgical procedure, involving two different incisions while aiming to remove the specimen. In order to decrease the invasivity of the operation, a large variety of endoscopic procedures were introduced, with the purpose of approaching the intramural ureter and detaching it from the bladder.

Two large categories of these techniques emerged during the last 50 years: stripping (intussusception), in which the segment is removed antegradely, and pluck technique, or retrograde removal of the ureter.

The use of bipolar plasma vaporization in prostatic and bladder pathology emphasized promising results with regard to safety, visibility and local control. Based on these characteristics, we used it during ureteral detachment.

The aim of our study was to evaluate the efficacy and safety of bipolar perimeatal plasma vaporization in saline during the pluck technique.

## Materials and methods

During the last two years, nephroureterectomy following bipolar plasma vaporization – pluck transurethral detachment of the intramural ureter was performed in 42 cases.

We used the Olympus SurgMaster UES-40 bipolar generator (Olympus, Tokyo, Japan), saline continuous flow irrigation and a “button” type vapo-resection electrode (Olympus, Hamburg, Germany).

**Fig. 1:**
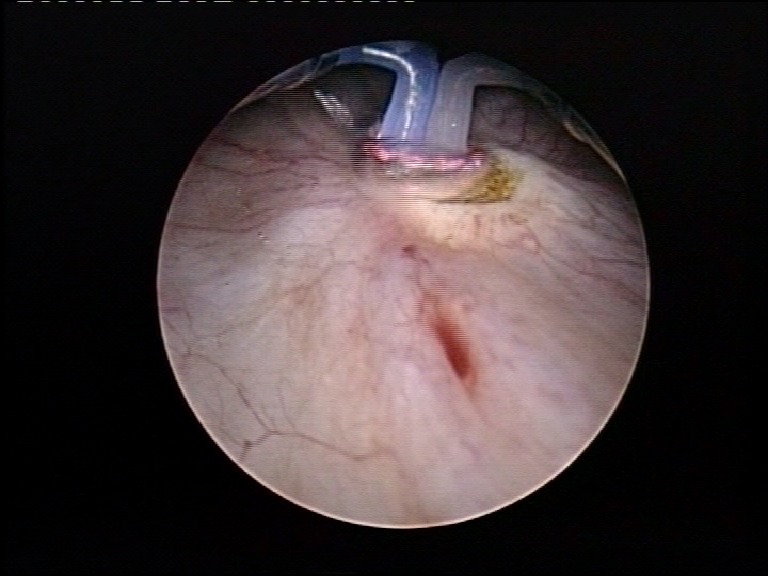
Beginning of the perimeatal vaporization, at 12 o’clock

The procedure started with the patient in standard lithotomy position. The perimeatal vaporization started at 12 o’clock, 1 centimeter away from the ureteral orifice (**Fig. 1**). 

**Fig. 2:**
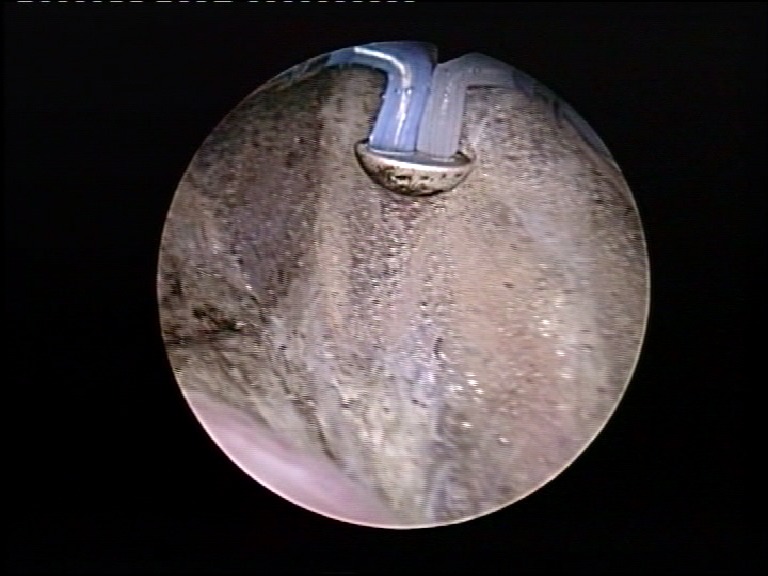
Mechanical dissection of the intramural ureter

Afterwards, the vaporization circumscribed it, until the perivesical fat was clearly exposed. In order to facilitate the detachment, further mechanical dissection using the “button” electrode was performed at the end of the procedure (**Fig. 2**). 

**Fig. 3:**
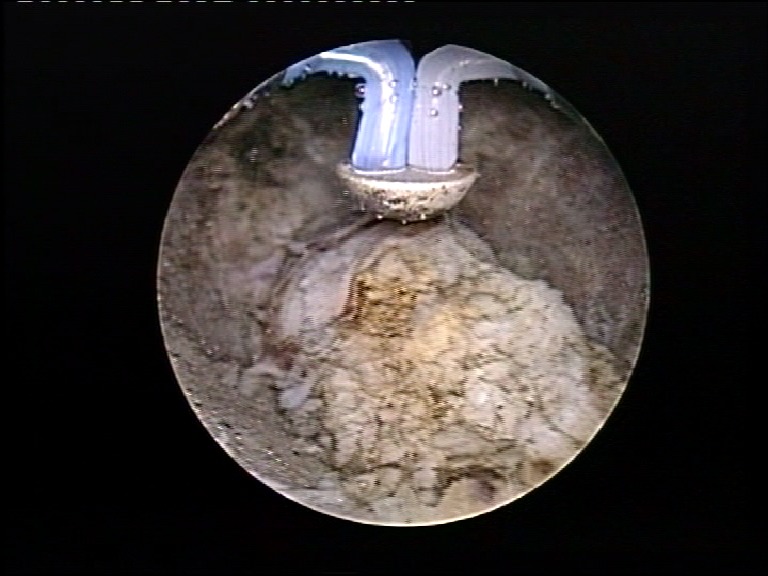
Coagulation of the ureteral orifice

In order to reduce the risk of tumor spillage, the ureteral orifice was thoroughly coagulated (**Fig. 3**). A 20F Foley catheter was indwelled in each case.

**Fig. 4:**
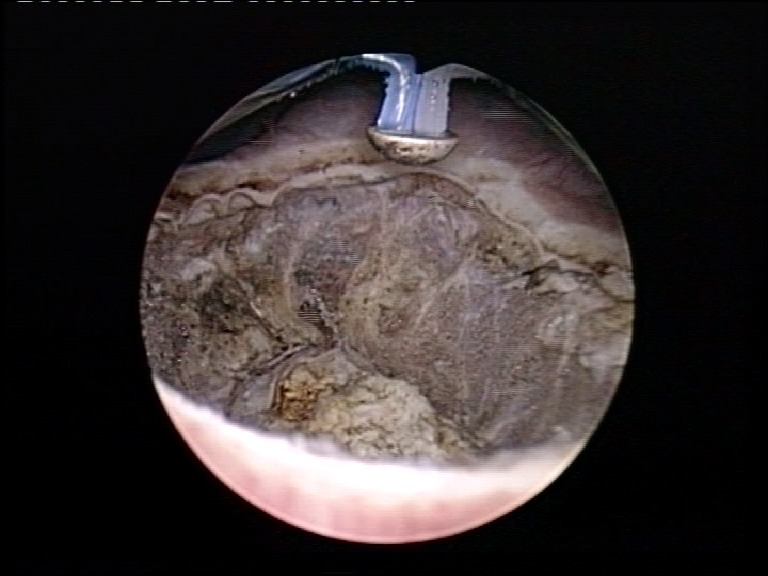
Complete circumferential detachment of the intramural ureter from the bladder wall

After detachment (**Fig. 4**), the patients were placed in lateral decubitus for transcostal lumbotomy. The ureter was ligatured under the presumed position of the tumor. 

**Fig. 5:**
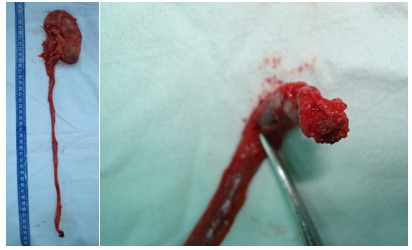
Nephroureterectomy specimen, with identifiable coagulated ureteral orifice

Digital dissection of the ureter was performed until achieving the extraction of the distal end from the bladder wall. Recognition of the coagulated ureteral orifice confirmed the removal of the entire upper urinary tract (**Fig. 5**). Subsequently, the kidney was dissected, the pedicle was ligatured and sectioned and the entire specimen was removed “en bloc”. 

The tumors were pyelocaliceal in 34 cases, ureteral in 7 cases and synchronous ureteral and pyelocaliceal in one case. The follow-up protocol included urinary cytology, ultrasonography, intravenous pyelography and CT

## Results

All the procedures were successfully completed. 

The detachment was performed while using a reduced irrigation flow, in order to minimize the perivesical fluid extravasation. Due to the good haemostatic capabilities, the visibility remained excellent throughout the procedure. 

Subjectively, the bipolar plasma vaporization allowed a very good local control. It permitted the muscular fibers to be clearly distinguished from the perivesical tissues, thus allowing a very precise detachment to be achieved.

The mean endoscopic detachment time was of 15 minutes for the entire series, 21 minutes for the first 10 cases, 14 minutes for the next 10 cases and 11 minutes for the last 12 cases.

All the postoperative specimens had the coagulated ureteral orifice at the distal end, thus confirming the removal of the entire ureter.

**Fig. 6:**
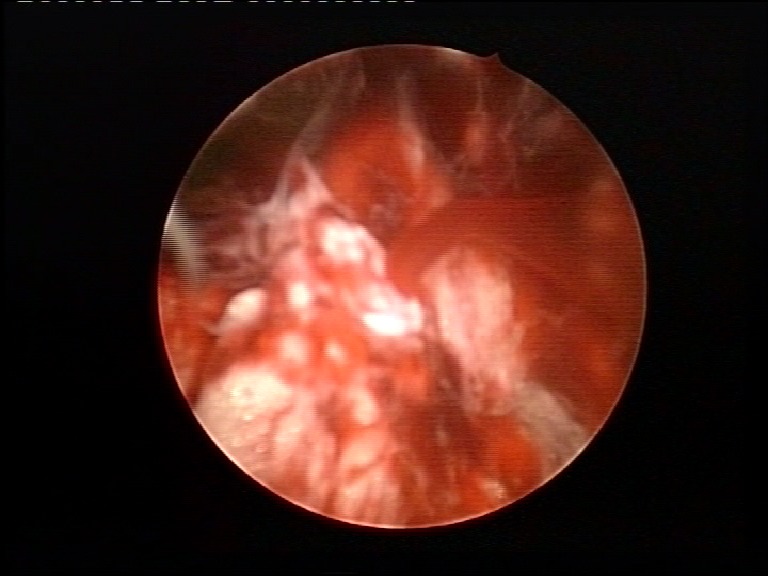
Active bleeding source on the detachment area

**Fig. 7:**
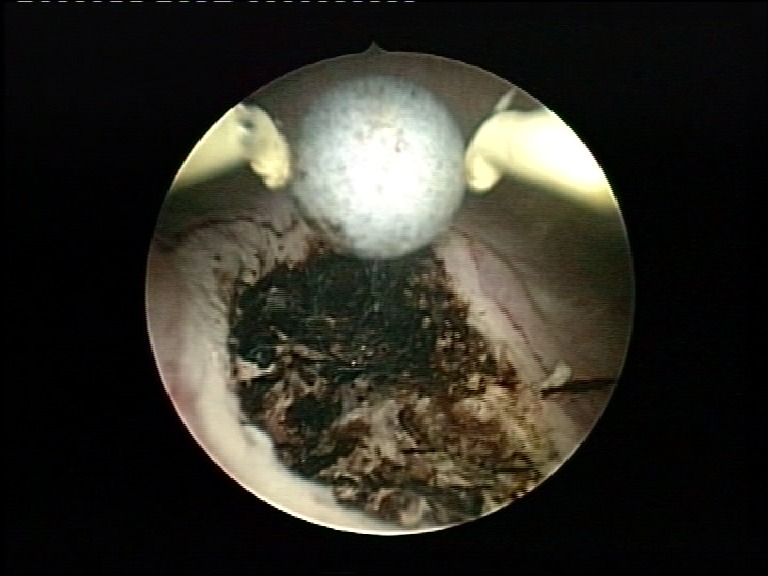
Coagulation of the detachment area for haemostatic purposes

The complication rate was of 4.8% and consisted of 2 cases of postoperative hematuria. One of them was managed conservatively, while the other imposed endoscopic re-intervention in the first day after the initial procedure. An active bleeding source located at the margins of the detachment area was coagulated in this case (**Fig. 6,7**).

Regarding the tumor stage, 16 patients presented stage pTa, 10 stage pT1, 9 stage pT2 and 7 were diagnosed with stage pT3.

The mean follow-up period was of 14 months (ranging between 8 and 26 months). During the follow-up, six patients emphasized bladder recurrences (none situated on the detachment area), one presented renal fossa tumor and one had secondary lymph-node invasion. The disease-specific mortality was of 4.8%.

## Discussion

Upper urinary tract urothelial tumors account for approximately 5% of all urothelial cancers and 10% of all renal malignancies [**1**]. Several reasons were advocated for the use of nephroureterectomy with bladder cuff removal as standard treatment of this pathology: high recurrence rate in the distal ureter (16-58%), significant likelihood of multicentricity on the same side (15-44%) and low incidence of bilateral tumors (2-5%) [**2,3**]. 

However, the standard open technique implies either two incisions (lumbar and lower midline abdominal) or an extensive lumbo-abdominal incision, both methods causing considerable trauma to the abdominal wall and, therefore, prolonged postoperative pain.

Consequently, large varieties of endoscopic procedures for ureteral detachment during nephroureterectomy were described in the literature. To our knowledge, this is the first report of using bipolar plasma vaporization to isolate the intramural ureter from the bladder wall.

The literature data described complication rates for the pluck technique varying between 3.2% and 12.5%. Most of these cases presented perivesical extravasation of urine [**4,5,6**], a complication which was not encountered in our series. A possible explanation for this aspect may be represented by the significantly lower irrigation flow specific to the bipolar vaporization when compared to the monopolar approach. Excellent visibility and good local control of the tissues also contributed to a more precise detachment of the ureter.

Compared to the ureteral stripping, the pluck technique presented the advantage of the “en bloc” removal of the entire upper urinary tract, while repositioning the patient a single time. Due to this feature, the method can be applied not only in pyelocaliceal tumors, but also for the ureteral locations.

However, there are also certain advantages of the stripping technique. One of them is represented by the reduced risk of tumor cells spillage. Nine cases of bladder recurrences in the resected area were reported after pluck detachment by comparison to none in patients in whom stripping was applied [**7**]. In order to reduce this risk of spilling, coagulation of the ureteral orifice was recommended by many authors, a technical artifice that we also applied in each case. Furthermore, a ligature may be performed during the open surgical part, lower than the presumed tumor site. In our cases, we encountered no bladder recurrences on the resection site and no pelvic recurrences. These data proved the safety of the pluck technique, with minimal risk of tumor cells spillage during detachment.

With the above-mentioned safeguards taken into consideration, the pluck technique seems to be applicable with no significant risk even in cases of proximal ureteral tumors [**7,8**]. In our experience, the pluck technique was safely performed in ureteral tumors.

The bladder recurrences occur in open series in 25-30% of the cases, usually during the first 2-3 years after surgery [**2,9-11**]. In a meta-analysis involving 129 cases of pluck detachment, Laguna and De La Rosette reported a similar rate of bladder recurrences, respectively 24% [**7**]. In our study, this type of recurrence was encountered in 12.5% of the patients. 

The endoscopic approach of the intramural ureter significantly reduced the operative times compared to the open surgical technique. Among the various minimally invasive procedures, the reported operative times for the transurethral part varied between 30 minutes for pluck desinsertion using monopolar resection [**12,13**] and one hour when the ureteral orifice was also ligatured using an endo-loop [**6**]. For the new technique of bipolar vaporization detachment, the mean endoscopic operative time determined was of 14 minutes. In addition, the dynamics of mean detachment times demonstrated a short learning curve. 

The 14 months follow-up period emphasized satisfactory oncological outcomes.

## Conclusions

The endoscopic approach of the terminal ureter using bipolar plasma vaporization as part of one-step nephroureterectomy is a safe and effective method.

The excellent intraoperative visibility and good local control made this procedure facile and with a short learning curve.

Pluck ureteral detachment proved to have good oncological results, at least when some safeguards (designed to reduce tumor cells spillage) are observed.
